# Encephalomyelitis Caused by *Balamuthia mandrillaris* in a Woman With Breast Cancer: A Case Report and Review of the Literature

**DOI:** 10.3389/fimmu.2021.768065

**Published:** 2022-01-05

**Authors:** Juan Hu, Yiqi Zhang, Yongwei Yu, Huili Yu, Siruo Guo, Ding Shi, Jianqin He, Chi Hu, Jiqi Yang, Xueling Fang, Yonghong Xiao

**Affiliations:** ^1^ Intensive Care Unit, The First Affiliated Hospital, Zhejiang University School of Medicine, Hangzhou, China; ^2^ State Key Laboratory for Diagnosis and Treatment of Infectious Diseases, National Clinical Research Center for Infectious Diseases, Collaborative Innovation Center for Diagnosis and Treatment of Infectious Diseases, The First Affiliated Hospital, Zhejiang University School of Medicine, Hangzhou, China; ^3^ Department of Neurosurgery, The First Affiliated Hospital, Zhejiang University School of Medicine, Hangzhou, China

**Keywords:** encephalomyelitis, *Balamuthia mandrillaris*, next-generation sequencing, T-cell receptor, encephalitis

## Abstract

*Balamuthia mandrillaris* is one cause of a rare and severe brain infection called granulomatous amoebic encephalitis (GAE), which has a mortality rate of >90%. Diagnosis of *Balamuthia* GAE is difficult because symptoms are non-specific. Here, we report a case of *Balamuthia* amoebic encephalomyelitis (encephalitis and myelitis) in a woman with breast cancer. She sustained trauma near a garbage dump 2 years ago and subsequently developed a skin lesion with a *Mycobacterium abscessus* infection. She experienced dizziness, lethargy, nausea and vomiting, inability to walk, and deterioration of consciousness. Next-generation sequencing of cerebrospinal fluid (CSF) samples revealed *B. mandrillaris*, and MRI of both brain and spinal cord showed abnormal signals. T-cell receptor (TCR) sequencing of the CSF identified the Top1 TCR. A combination of amphotericin B, flucytosine, fluconazole, sulfamethoxazole, trimethoprim, clarithromycin, pentamidine, and miltefosine was administrated, but she deteriorated gradually and died on day 27 post-admission.

## Introduction

*Balamuthia mandrillaris*, an emerging opportunistic protozoan pathogen, is one of four free-living amoebae that can infect humans; the pathogen causes granulomatous amoebic encephalitis (GAE), which is often fatal. The clinical symptoms are similar to those of viral or bacterial meningitis (i.e., headache, stiff neck, fever, and photophobia). The incubation period is unclear, but it usually follows a subacute to chronic course lasting from months to years; however, when it progresses to brain involvement, death is common (1–3). Indeed, the low morbidity, difficulty of diagnosis, and lack of proven effective treatments (4) mean that, to date, only about 10/200 patients with *Balamuthia* GAE have survived central nervous system (CNS) invasion (5–7), resulting in a mortality rate of >90% (8). Here, we report the first case of *Balamuthia* amoebic encephalomyelitis (encephalitis and myelitis) in a patient with a history of breast cancer and a *Mycobacterium abscessus* infection.

## Case Description

**Figure 1:**
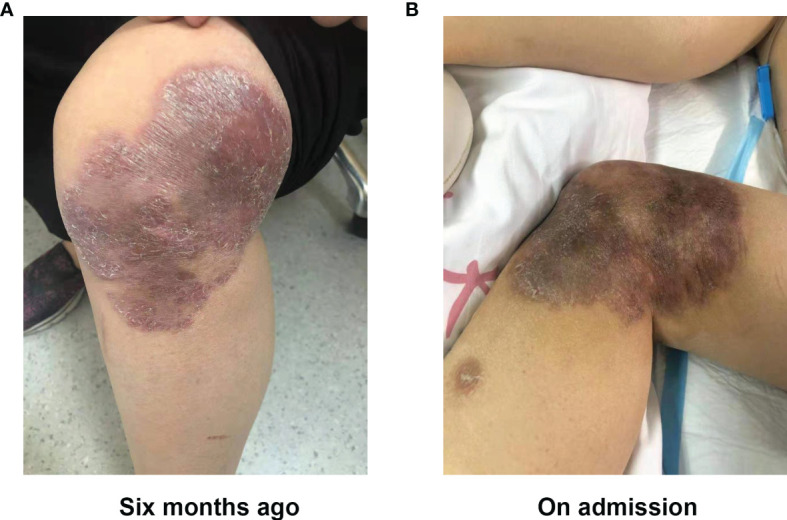
Skin lesion of the left knee 6 months before **(A)** and on admission **(B)**.

A 37-year-old women, who underwent radical mastectomy for cancer of the left breast 1 week previously, visited the local hospital due to dizziness. After receiving symptomatic treatment, she experienced clinical deterioration, with lethargy, nausea, and vomiting, and she was unable to walk. There was no fever. Brain magnetic resonance imaging (MRI) showed abnormal intracranial signals in multiple areas. Four days later, she attended the neurology department of our hospital to check for intracranial metastases. A neurological examination revealed nuchal rigidity, eyes gazing to the right, loss of muscle strength in the left arm and leg (strength was 2/5 in both), and a positive Pap sign on the right. Other physical examinations revealed a dark red desquamation infection lesion (about 15 × 10 cm) on the left knee (**Figures 1A, B**). There was no medical history of severe infections, failure to thrive, eczema, chronic diarrhea, unexplained fevers, radiotherapy, or chemotherapy. She denied recent travel, any history of freshwater swimming, or consumption of uncooked meats or unpasteurized dairy products. However, she suffered trauma 2 years ago; she sustained a wound to the left lower limb after a fall near a garbage dump, which healed poorly. Six months previously, she developed a skin infection of the left limbs. Histopathological examination showed diffuse inflammatory cell infiltration of the dermis, with granuloma formation and multinucleate giant cells. Next-generation sequencing (NGS) revealed *M. abscessus*. A diagnosis of *M. abscessus* infection led to a combined therapy with rifampicin (450 mg, QD), ethambutol (0.75 g, QD), colabitol (500 mg, QD), and clarithromycin (500 mg, BID). Combined with the results of neurological examination and the presence of the skin lesion, a diagnosis of CNS *M. abscessus* infection was considered, and the previous therapy was continued. Mannitol infusion was instigated to reduce intracranial pressure.

Analysis of the cerebrospinal fluid (CSF) revealed the following: karyocytes, 310/μl (8% neutrophilic granulocytes and 88% lymphocytes); red cells, 30/μl; protein, 1.515 g/L (normal range, 0.150–0.450 g/L); glucose, 1.7 mmol/L (normal range, 2.5–4.5 mmol/L); and chloride, 115 mmol/L (normal range, 120–131 mmol/L). Intracranial pressure was 330 mmH_2_O. Routine laboratory values, including a complete blood count, procalcitonin, and C-reactive protein, were within normal limits. Microscopic examination with India ink staining, bacterial and *Mycobacterium tuberculosis* cultures, and cerebrospinal cytology were all negative. Brain MRI revealed multifocal enhanced mass-like lesions (in the right temporal lobe, right thalamus, left parahippocampal gyrus and pontine, and fourth ventricle) with enhancement and edema. Her condition continued to worsen and she developed headache, fever, and somnolence. Neurological examination showed that the muscle strength in the right limbs had weakened further. After consultation with infectious disease specialists, she received isoniazid (0.3 g, QD), pyrazinamide (500 mg, TID), and linezolid (600 mg, Q12H) to treat suspected *M. abscessus* infection.

**Figure 2:**
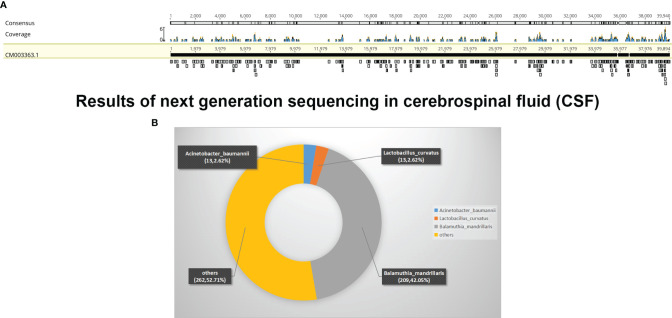
Results of next-generation sequencing in cerebrospinal fluid (CSF). **(A)** mNGS result of nucleotide sequences distributed along the genome of *Balamuthia mandrillaris* in the case. **(B)** Read composition of microbes in the CSF sample.

On day 9 of hospitalization, she was transferred to the ICU after tracheal intubation due to deterioration of consciousness (GCS = 10 points) and decreased oxygenation. Emergency brain CT revealed dilated ventricles and reduced brainstem density. We performed left lateral ventricular drainage, a routine microscopic examination (including India ink staining), bacterial and *M. tuberculosis* cultures, and cerebrospinal cytology; all were negative. Fortunately, NGS identified *B. mandrillaris* (**Figures 2A, B**). Based on the available information, including the specific NGS outcome, the history of trauma, and the skin lesion, she was diagnosed with GAE caused by *B. mandrillaris.* She was treated with amphotericin B (40 mg, QD), flucytosine (500 mg, TID), fluconazole (800 mg, QD), sulfamethoxazole and trimethoprim (1.44 g, QID), clarithromycin (500 mg, QD), pentamidine (300 mg, QD), and intravenous immunoglobulin (20 g, QD).

**Figure 3:**
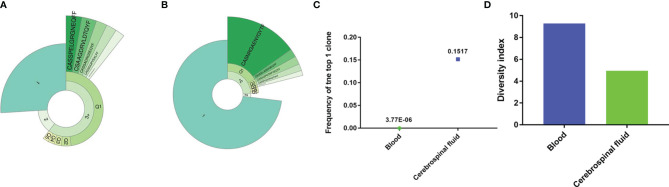
The TCR repertoire in blood and cerebrospinal fluid. The donut chart of the blood **(A)** and cerebrospinal fluid **(B)** sample from the patient displays the T-cell clonal expansion in the cerebrospinal fluid. The fan-shaped area displays the corresponding clonal frequency. The radian of 1, 2, and 3+ represented the total frequency of TCR sequences having 1, 2, and 3 or more reads in the sample, respectively. The top 5 clones’ amino acid sequences of complementary determination region 3 (CDR3) of TCR were shown in the donut chart, and the radian of the region, which was marked with the amino acid sequence, demonstrated the frequency of the corresponding T-cell clones. The larger radian means higher frequency. **(C)** The frequency of the top T-cell clones in the cerebrospinal fluid and blood samples. **(D)** The diversity of T-cell repertoire in both peripheral blood and cerebrospinal fluid.

During treatment, her lymphocyte count and immune cell subpopulations were within the normal range. To test the ability of T cells to recognize infections, we performed T-cell receptor (TCR) sequencing using peripheral blood and CSF samples. The Top1 TCR in the CSF sample showed high clonal proliferation (**Figures 3A, B**), with a cloning frequency of 15%; the same CDR3 amino acid (AA) sequence CASNRGAENYGYTF was also detected in the peripheral blood, although with a frequency of 3.77E-06 (**Figure 3C**). At the same time, we compared the TCR diversity in the CSF and peripheral blood. The TCR diversity in the CSF was significantly lower than that in peripheral blood (**Figure 3D**), which may be related to the lower number of lymphocytes in the CSF.

**Figure 4:**
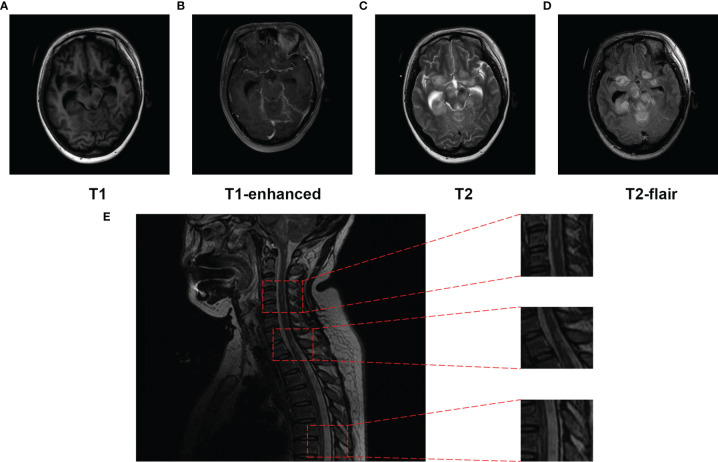
Magnetic resonance imaging (MRI) of the brain performed and spinal cord (T2) on day 15 post-hospitalization. **(A–D)** The frontal lobes, temporal lobes, ventriculus lateralis, brainstem, cisterna, and cerebellum were displayed as multiple patches, with high signal on T2WI, low signal on T1WI, apparently high signal on T2-Flair, and T1 enhanced. **(E)** Punctate and patchy lesions were presented as high T2 signals in the cervical and thoracic spinal cord.

On day 15 post-admission, the patient’s consciousness deteriorated further, with a GCS score of 1+T+1, and brain MRI showed that the multifocal enhanced mass-like lesions were more numerous than before (**Figures 4A–D**). MRI of the cervical thoracic segment revealed that the spinal cord functioned naturally, with punctate and patchy longer T2 signals, which were vaguely enhanced (**Figure 4E**). *Balamuthia* encephalomyelitis was diagnosed, and she was treated with intrathecal injection of amphotericin B and miltefosine (50 mg, TID) on hospital day 16. However, she experienced rapid clinical deterioration and spontaneous breathing stopped. On day 27 post-admission, the woman’s family requested a transition home to allow a natural death.

## Discussion

Here, we report a female patient with breast cancer who suffered trauma near a garbage dump 2 years prior to attending our hospital. She presented to our hospital with non-specific symptoms; however, NGS identified *Balamuthia* infection, and MRI revealed brain and spinal cord involvement. TCR sequencing identified Top1 TCR in both CSF and peripheral blood. Eventually, the patient died, despite receiving treatment with multiple drugs.

*B. mandrillaris*, a free-living amoeba, was first identified in the brain of a pregnant mandrill baboon that died of encephalitis in 1986 (9). The first report of amoebic meningoencephalitis caused by *Balamuthia* infection was published in 1990 (9). Unlike other amoeba, which are ubiquitous in water, soil, air, sewage, swimming pools, flowerpots, water tubs, humidifiers, aquaria, eye wash solutions, and hospital environments (1, 10), *B. mandrillaris* is difficult to isolate and culture. Its life cycle has two stages: trophozoites (representing the infective stage), which replicate by mitosis; and cysts, which are highly resistant to physical and chemical agents, and represent the dormant stage that protects the parasite (11). Cysts enable invasion of the host pulmonary system (12), nasal/olfactory nerve (13), skin (14), and possibly the gastrointestinal system (15). From there, they can spread to the brain. In humans, they can cause disease in both healthy and immunosuppressed individuals, particularly the young and the elderly (16). After sustaining a skin lesion on the face, trunk, or limbs, *Balamuthia* infection can cause a wide range of symptoms and then progress to the brain. Unfortunately, the symptoms are not specific to GAE. Early symptoms might include fever, headache, vomiting, lethargy, and nausea; after progression to the brain, other non-specific symptoms include mental health changes, seizures, weakness, confusion, and partial paralysis. Due to low morbidity, difficulty in diagnosis, and lack of a proven effective treatment, GAE is a significant threat to human health, with a 90% death rate (17).

Risk factors for GAE remain unclear; exposure to an environment in which *B. mandrillaris* is distributed widely, another primary infection, an underlying genetic factor, or a combination of the above may be potential sources (3). Evidence suggests that Hispanics individuals comprise a large proportion of *Balamuthia* patients (8, 18). An additional and troubling situation has been reported recently in the USA: the CDC reported that it is possible to acquire the infection after organ transplantation (19, 20). Cysts or trophozoites enter the body through a lesion in the skin or by inhalation, after which they invade the bloodstream and spread hematogenously to the brain or through olfactory nerve structures (1, 3, 21). Recent studies in animal models show that the gastrointestinal tract is also a possible route of entry (15). Typically, a skin lesion is a painless plaque or ulcer in the center of the face or on an extremity (especially the knee) (22); studies in Peru show that such a lesion was present in almost all affected patients (5, 22, 23). Here, we describe a female patient who was exposed to trauma near a garbage dump 2 years ago. Her left lower limb (knee joint) was injured, and the wound did not heal, leading to development of an infectious skin lesion 6 months ago. Although a previous *M. abscessum* infection was diagnosed and there was no evidence of amoebae in the skin tissue slices, skin histopathology at the time revealed granulomatous inflammation, which is consistent with the outcome of brain biopsies from *Balamuthia* GAE patients in another study (24). At present, we cannot rule out the possibility of a mixed infection. Furthermore, histopathological tests for amoebae are usually not positive, a factor that should be taken into consideration. Hence, we still consider that exposure to contaminated soil or water was a main predisposing factor in this case, and that breast cancer surgery may have accelerated disease progression.

In addition to GAE, *B. Mandrillaris* also causes skin lesions, and in a few cases, amoebae have been found in the kidneys, adrenal glands, pancreas, thyroid, and lungs (25–29). In these cases, the patients were also diagnosed with *Balamuthia* myelitis. Guarner et al. showed that both the trophozoite and cyst forms have characteristic nuclear morphology, and tend to cluster around blood vessels, which causes classic CNS angiitic lesions (30). This suggests the possibility of hematogenous spread to the brain and other organs. Hence, if a patient is suspected of being infected with *B. Mandrillaris*, a comprehensive assessment should be undertaken, and organs other than the brain should be biopsied. In this way, more GAE patents will be diagnosed.

One reason for the difficulty in diagnosing *Balamuthia* GAE is the non-specific symptoms, which are common to other pathogens that cause CNS infections; these include headache, stiff neck, fever, and photophobia. As GAE progresses, signs related to increased intracranial pressure, such as nausea, vomiting, personality changes, aphasia, acute confusion, seizures, and lethargy, become evident; lethargy progresses to coma and, ultimately, to death (1, 5, 21, 22). Amoebae are usually not found in the CSF, although they have been isolated from the CSF in a few cases (31, 32). Pleocytosis with lymphocytic predominance, normal or low glucose levels, and slightly to highly elevated protein levels are normally found in the CSF (33). Neuroimaging findings are also non-specific and often show lesions with surrounding edema, which would lead to misdiagnosis as a tumor, viral/bacterial or tuberculosis meningoencephalitis, acute disseminated encephalomyelitis, toxoplasmosis, or neurocysticercosis (1, 3, 34–36).

Lack of standard diagnostic tools also means that diagnosis of *Balamuthia* GAE is less likely. Currently, the most precise methods available include detection of amoebae in skin and brain tissue. However, brain biopsies are taken only during surgery or at autopsy, and the tissue tests either by immunofluorescence staining or by immunoperoxidase staining, which both use polyclonal rabbit anti-*Balamuthia* serum in paraffin-fixed tissue (5, 30), are only available in a few research centers. PCR and real-time PCR are also used to diagnose GAE (36, 37). Nowadays, NGS, a fast and accurate method for identification of pathogens, is helpful for diagnosis of unknown diseases; as such, it plays an important role in accurate diagnosis. In 2015, Wilson et al. detected *B. mandrillaris* encephalitis by metagenomic deep sequencing (38), and in 2020, Yang et al. reported the diagnosis of a 2-year-old Chinese boy with *B. mandrillaris*-related primary amoebic encephalitis by NGS (39). In the current case, the woman was also diagnosed with *Balamuthia* amoebic encephalomyelitis by NGS.

TCR is a heterodimeric protein located on the surface of mature T cells. It recognizes antigen peptides presented by major histocompatibility complex molecules and triggers a cellular immune response by T cells. Each mature T cell will express a specific sequence of αβ or γδ TCR on the surface. The two polypeptide chains of the TCR protein can be partitioned according to function. The chains comprise a variable region, a constant region, a transmembrane domain, and an intracellular region. The complementarity determining regions (CDR) in the variable region determine the target of antigen recognition. Because the polypeptide sequence and structure of this CDR region are highly variable, T cells can recognize different antigen molecules, which is one of the foundations of the body’s strong immunity. TCR sequencing of peripheral blood and CSF samples were performed in this case. The Top1 TCR, CDR3 AA sequence CASNRGAENYGYTF in CSF, was found in peripheral blood. It is speculated that T cells in the peripheral blood recognize this foreign antigen at an early stage; when the antigen enters the CSF, a strong T lymphocyte recognition stress occurs. Thus, we speculate that Top1 TCR might be disease-relevant and worthy of further investigation.

Treatment of *Balamuthia* GAE infection is not standardized. However, survivors usually received a combination of an azole (fluconazole or itraconazole), a macrolide antibiotic (azithromycin or clarithromycin), and pentamidine, sulfadiazine, flucytosine, thioridazine, and miltefosine (11, 40). Although the patient described in this article received all of the drugs listed above, she eventually died on day 27 post-admission. Thus, a novel antimicrobial drug that shows amoebicidal activity, good penetration through the blood–brain barrier, and minimal toxicity is required urgently.

*Balamuthia* GAE is gaining a reputation as a significant threat to human health due to a general lack of awareness, difficulty of diagnosis, and lack of an effective treatment. Thus, in addition to solving the above problems, we should take effective preventive measures to reduce occurrence of GAE, particularly reducing the exposure of immunocompromised people. Moreover, clinicians (especially those working in the community) should play a major role in educating patients about potential risks and how they can be mitigated.

## Data Availability Statement

The original contributions presented in the study are included in the article/supplementary material. Further inquiries can be directed to the corresponding authors.

## Ethics Statement

Written informed consent was obtained from the individual(s) for the publication of any potentially identifiable images or data included in this article.

## Author Contributions

JuH and YZ completed the main body of the manuscript. XF and YX made decisions about the entire treatment process and provided the main ideas for writing the final article. YY, HY, SG, DS, JiH, CH, and JY have supplemented and modified parts of the manuscript and participated in the collation of patient data. All authors contributed to the article and approved the submitted version.

## Conflict of Interest

The authors declare that the research was conducted in the absence of any commercial or financial relationships that could be construed as a potential conflict of interest.

## Publisher’s Note

All claims expressed in this article are solely those of the authors and do not necessarily represent those of their affiliated organizations, or those of the publisher, the editors and the reviewers. Any product that may be evaluated in this article, or claim that may be made by its manufacturer, is not guaranteed or endorsed by the publisher.
